# Supplementary material for: Online suicidal thoughts and/or behaviours talk: A scoping review protocol
Source: PLoS One. 2022 Oct 27;17(10):e0276776. doi: 10.1371/journal.pone.0276776 (PMC9612572; doi:10.1371/journal.pone.0276776)
Supplement: S1 File — (DOCX) [file pone.0276776.s001.docx]

**Supplementary File 1**

**Electronic Databases and their Associated Search Strings**

| **Database** | **String 1** | **String 2** | **String 3** | **String 4** | **Limiters** |
| --- | --- | --- | --- | --- | --- |
| EbscoHost Databases Academic Search Ultimate, APA PsycInfo, CINAHL with Full Text, Communication Source, Health Source: Nursing/Academic Edition, Humanities Source Ultimate, MLA Directory of Periodicals, Psychology and Behavioural Sciences Collection, Sociology Source Ultimate | internet OR “online bulletin board” OR “internet chat room” OR “online community” OR “online communities” “online social network” OR “online message board” OR “social media” OR Twitter* OR Facebook* OR Instagram* OR “insta gram” “online forum” OR web OR virtual OR “social networking site” OR sns OR mobile* OR computer OR myspace OR “mediated communication” OR forum* OR “online information services” OR “discussion forum” OR Snapchat* OR Weibo* | convers* OR talk* OR chat* OR interact* OR communicat* OR discuss* OR discourse OR discursive OR languag* | user OR member* OR poster | suicid* OR self-harm* OR self-injur* OR self-mutilation OR cybersuicide OR “self harm” OR self injur* or “ self mutilation” | peer reviewed journals  date range 1989 to present |
| Medline | internet OR “online bulletin board” OR “internet chat room” OR “online community” OR “online communities” “online social network” OR “online message board” OR “social media” OR Twitter* OR Facebook* OR Instagram* OR “insta gram” “online forum” OR web OR virtual OR “social networking site” OR sns OR mobile* OR computer OR myspace OR “mediated communication” OR forum* OR “online information services” OR “discussion forum” OR Snapchat* OR Weibo* | convers* OR talk* OR chat* OR interact* OR communicat* OR discuss* OR discourse OR discursive OR languag* | user OR member* OR poster | suicid* OR self-harm* OR self-injur* OR self-mutilation OR cybersuicide OR “self harm” OR self injur* or “ self mutilation” | No peer reviewed journal limitation required as all research included in MEDLINE is peer reviewed  date range 1989 to present |
| SAGE Journals: Social Sciences and Humanities | internet OR “online bulletin board” OR “internet chat room” OR “online community” OR “online communities” “online social network” OR “online message board” OR “social media” OR Twitter* OR Facebook* OR Instagram* OR “insta gram” “online forum” OR web OR virtual OR “social networking site” OR sns OR mobile* OR computer OR myspace OR “mediated communication” OR forum* OR “online information services” OR “discussion forum” OR Snapchat* OR Weibo* | convers* OR talk* OR chat* OR interact* OR communicat* OR discuss* OR discourse OR discursive OR languag* | user or member* or poster | suicid* OR self-harm* OR self-injur* OR self-mutilation OR cybersuicide OR “self harm” OR self injur* or “ self mutilation” | No peer reviewed journal limitation applied^%^  date range 1989 to present |
| Scopus | internet OR “online bulletin board” OR “internet chat room” OR “online community” OR “online communities” “online social network” OR “online message board” OR “social media” OR Twitter* OR Facebook* OR Instagram* OR “insta gram” “online forum” OR web OR virtual OR “social networking site” OR sns OR mobile* OR computer OR myspace OR “mediated communication” OR forum* OR “online information services” OR “discussion forum” OR Snapchat* OR Weibo* | convers* OR talk* OR chat* OR interact* OR communicat* OR discuss* OR discourse OR discursive OR languag* | user or member* or poster | suicid* OR self-harm* OR self-injur* OR self-mutilation OR cybersuicide OR “self harm” OR self injur* or “ self mutilation” | No peer reviewed journal limitation applied%  date range 1989 to present |

Notes: ^date range and ^%^peer review journal will be applied as inclusion-exclusion criteria during step 3 as these databases do not allow this limitation to be imposed during the search process
