# Supplementary material for: Online suicidal thoughts and/or behaviours talk: A scoping review protocol
Source: PLoS One. 2022 Oct 27;17(10):e0276776. doi: 10.1371/journal.pone.0276776 (PMC9612572; doi:10.1371/journal.pone.0276776)
Supplement: S4 File — (DOCX) [file pone.0276776.s004.docx]

**Supplementary File 6**

Rating Rules for Assessing Quality of Measures

| **Rating** | **Reason** |
| --- | --- |
| **H** | - Objective measures |
|  | - Multi-item validated measure |
| **M/H** | - Single item from validated measure (which recently has had a validity study done for suicidality items) |
|  | - Written by researchers and adequate validity and reliability data reported |
| **M** | - Adapted from validated scales -- might be close but some words slightly changed with no apparent problems in the change |
|  | - Written by researchers and adequate reliability reported but no validity |
|  | - Extensive measure written by researchers when nothing else exists to measure the construct, even if no validity/reliability |
| **M/L** | - Short measure written by researchers and no reliability/validity info reported and no apparent problems present |
| **L** | - Validated measure but problems present (e.g., outdated measure, unclear items, items mix concepts) |
|  | - Written by researchers and problems may be present (e.g., unclear wording, stigmatizing language) |

Note: H = high, M/H = medium/high, M = medium, M/L = medium/low, L = low
