# Supplementary figures and images for: Online suicidal thoughts and/or behaviours talk: A scoping review protocol
Source: PLoS One. 2022 Oct 27;17(10):e0276776. doi: 10.1371/journal.pone.0276776 (PMC9612572; doi:10.1371/journal.pone.0276776)

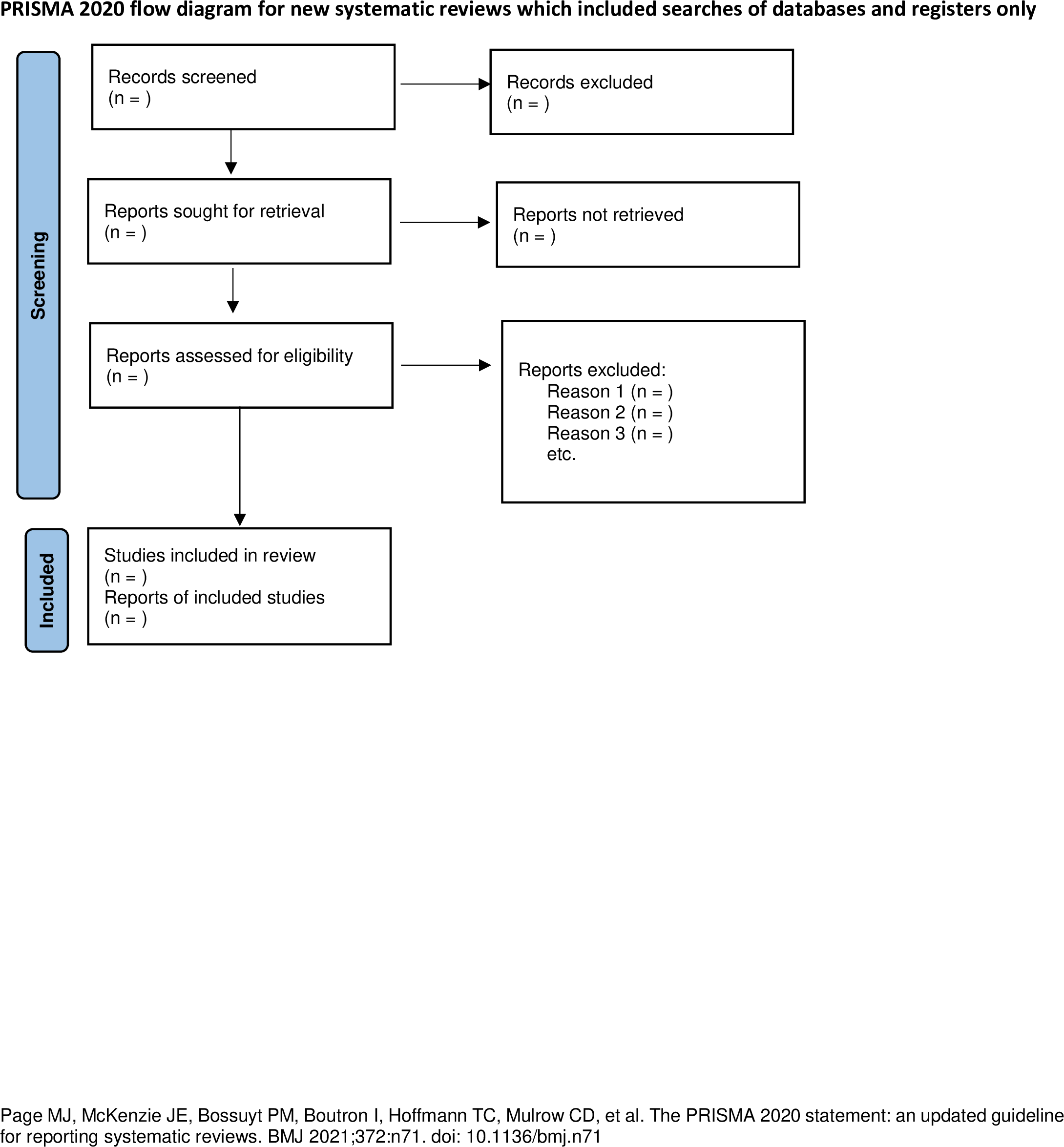

Supplement: S1 Fig — (ZIP) [file pone.0276776.s006.zip › S1b_Fig.tif]

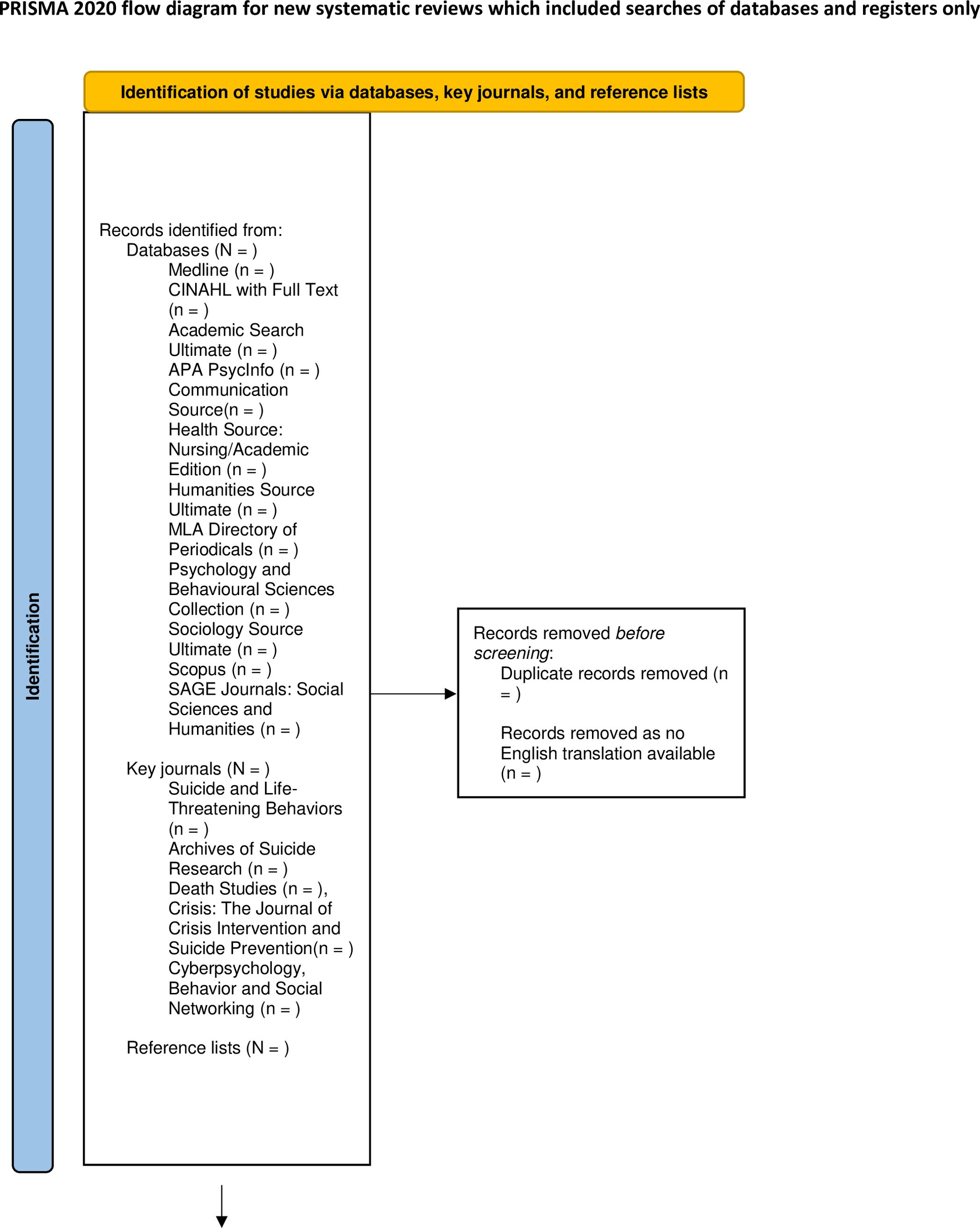

Supplement: S1 Fig — (ZIP) [file pone.0276776.s006.zip › S1a_Fig.tif]
